# Toxic Effects of Waterborne Nitrite on LC_50_, Hematological Parameters, and Plasma Biochemistry in Starry Flounder (*Platichthys stellatus*)

**DOI:** 10.3390/toxics13090748

**Published:** 2025-09-02

**Authors:** Bijae Gong, Hyeong Su Kim, Cheol Young Choi, Sung-Pyo Hur, Jun-Hwan Kim

**Affiliations:** 1 Department of Marine Life Science, Jeju National University, Jeju 63243, Republic of Korea; qlwo0907@stu.jejunu.ac.kr (B.G.); hursp@jejunu.ac.kr (S.-P.H.); 2 Aquaculture Research Division, National Institute of Fisheries Science, Busan 46083, Republic of Korea; kimk2k@korea.kr; 3 Division of Marine BioScience, National Korea Maritime and Ocean University, Busan 49112, Republic of Korea; 4 Department of Aquatic Life Medicine, Jeju National University, Jeju 63243, Republic of Korea

**Keywords:** *Platichthys stellatus*, nitrite exposure, LC_50_, hematological parameters, plasma components

## Abstract

Nitrite is a common environmental pollutant in aquaculture systems, where high levels can severely impair fish physiology and survival. This study aimed to evaluate the acute toxicity of waterborne nitrite in starry flounder (*Platichthys stellatus*). Fish (mean weight 145.69 ± 16.06 g, mean total length 22.78 ± 0.70 cm) were exposed to nitrite concentrations of 0, 25, 50, 100, 200, 400, and 800 mg NO_2_^−^/L for 96 h. The lethal concentration 50 (LC_50_) of nitrite for *P. stellatus* was determined to be 574.47 mg NO_2_^−^/L. Hematological parameters such as red blood cell counts (RBCs), hemoglobin (Hb), and hematocrit (Hct) were significantly decreased by nitrite exposure. Plasma components including calcium (Ca^2+^), glucose, cholesterol, aspartate aminotransferase (AST), and alanine aminotransferase (ALT) were significantly changed by nitrite exposure. The results of this study suggest that acute exposure to waterborne nitrite (>200 mg NO_2_^−^/L) adversely affects survival rates, hematological parameters, and plasma components in *P. stellatus*. These findings provide important baseline data for nitrite toxicity assessment in *P. stellatus*.

## 1. Introduction

Nitrite is one of the common nitrogen compounds in the natural aquatic environment; it is an intermediate product in the nitrification process of ammonia generated during metabolic processes in aquatic animals into nitrate by bacteria [1]. Nitrite occurs naturally during metabolic processes in aquatic organisms, but high levels of nitrite are also widely used in the food and chemical industries as well as agricultural fertilizers, which can enter the aquatic ecosystem through wastewater due to its high water solubility [2]. Nitrite is generally present at low concentrations in the aquatic ecosystem; it has a short residence time in the natural environment and normal aquaculture environments, because nitrite is rapidly oxidized in an environment with sufficient oxygen [3]. However, nitrification may be hindered by various water environmental factors such as water temperature, pH, and dissolved oxygen [4]. In addition, the reduction in nitrates can also result in high levels of nitrite exposure in aquatic ecosystems, and exposure to nitrite above the acceptable limit can be fatally toxic to aquatic organisms [5].

High levels of nitrite can cause growth inhibition, disruption of physiological responses, and oxidative damage to aquaculture fish, and this imbalance in homeostasis can lead to functional impairment and death due to environmental diseases [6,7]. Additionally, fish exposed to high waterborne nitrite become vulnerable to pathogenic bacteria or viruses due to their increased susceptibility to disease [8]. In general, nitrite (NO_2_^−^) can act competitively with chlorine (Cl^−^) due to its electronic similarity, which induces disturbances in the ionic balance in fish, thereby affecting cell signaling [9]. Nitrite can be actively absorbed into the fish body through the gills, and methemoglobinemia, a condition in which hemoglobin is converted into methemoglobin that lacks oxygen-carrying capacity, is a representative symptom of nitrite toxicity [10]. In addition, continuous exposure to nitrite can cause accumulation of nitrite in the fish body, resulting in changes in the histological structure of lysosomes, mitochondria, and organelles, which can lead to death due to functional impairment [11]. In other words, nitrite exposure can cause tissue hypoxia, disruption of physiological functions and disturbance of acid–base balance in fish, as well as protein degeneration due to oxidative damage [12].

In aquatic ecosystems, high levels of nitrite exposure are highly toxic to fish and lead to mass mortality in aquatic animals by affecting various physiological functions such as respiratory, ionoregulatory, endocrine, and cardiovascular functions [13,14]. In particular, mass mortality can occur when nitrite concentrations exceed the tolerance limit of fish [15], and it is very important to confirm the concentration–response relationship in order to evaluate nitrite toxicity to fish [16]. The lethal concentration 50% (LC_50_) refers to the concentration at which 50% of animals exposed to a specific toxicant die, and is widely used as a major indicator of acute toxicity assessment [17]. In ecotoxicity tests, the survival of experimental animals has important biological and ecological meanings; LC_50_ values determined from nitrite exposure can provide a baseline indicator to determine the tolerance limits in fish to nitrite and assess the hazards of nitrite in aquatic ecosystems [18,19].

Nitrite can enter the blood circulation through the gill ionocytes of fish, where it can accumulate to high levels in the blood plasma; hematological parameters and plasma components are considered the primary targets in fish exposed to waterborne nitrite [20]. Nitrite accumulation in the blood of fish can convert hemoglobin in red blood cells into methemoglobin, which cannot carry oxygen, thereby inducing symptoms of oxygen deficiency [21]. Disturbance of gill ion exchange, tissue hypoxia, and methemoglobinemia in fish due to nitrite exposure may lead to changes in blood oxygen transport and respiratory properties; this leads to disturbances in acid–base status and electrolyte balance [22]. Tissue accumulation of nitrite due to nitrite exposure disrupts K^+^ homeostasis and can be lost from red blood cells and skeletal muscle, causing extracellular hyperkalemia [23]. Hematological parameters and plasma components are major indicators for evaluating the health status in fish, and they can be used as reliable indicators for evaluating the toxic effects of exposure to harmful substances, as well as nutritional deficiencies and water quality deterioration [24]. Therefore, changes in the hematological parameters and plasma components in fish due to nitrite exposure can provide major information for understanding and evaluating the toxic physiology in fish by waterborne nitrite exposure.

Starry flounder, *Platichthys stellatus* belongs to the Pleuronectidae family of the Pleuronectiformes order and is a cold-water fish species distributed in the North Pacific [25]. It mainly lives near shallow coastal waters, but it can also be found in brackish and freshwater areas, showing its adaptability to a wide range of salinity [26]. Due to its adaptability to a wide range of habitats and high disease resistance, *P. stellatus* is currently used as a major aquaculture species in Korea [27]. Meanwhile, recent eco-friendly aquaculture techniques such as recirculating aquaculture systems (RAS) and bio-floc aquaculture are closed aquaculture systems that may contain high concentrations of nitrite, and high levels of nitrite exposure can be fatal to aquaculture organisms [28]. The toxic effects and tolerance limits of nitrite have been extensively studied in freshwater species, but research on marine fish remains limited. Due to differences in osmoregulatory physiology, marine fish may exhibit greater resistance to nitrite toxicity than freshwater species [4]. However, little research has been conducted on the tolerance limits and toxic effects of nitrite exposure in *P. stellatus*. In particular, understanding species-specific nitrite toxicity in *P. stellatus* is essential to ensure animal welfare and to safely extend the transport distance of live fish. Therefore, the purpose of this study was to suggest indicators of nitrite tolerance limits and toxicity criteria by analyzing the lethal concentration and hematological changes in *P. stellatus* following acute nitrite exposure.

## 2. Materials and Methods

### 2.1. Experimental Fish and Environment

*P. stellatus* (mean weight 145.69 ± 16.06 g, mean total length 22.78 ± 0.70 cm) was obtained from a fish farm in Seogwipo, Jeju. Prior to the experiment, the fish were acclimated for 2 weeks in 700 L polycarbonate tanks at a stocking density of approximately 20.8 kg/m^3^, at 17.0 ± 0.5 °C and 6.09 ± 0.16 mg/L dissolved oxygen. The fish were fed a commercial diet twice daily (3:00 p.m. and 9:00 p.m.) at 3% of body weight per day. Uneaten feed was removed within 10 min after feeding. Following acclimation, a total of 42 fish (6 fish per group) were subjected to acute 96 h exposure to 7 nitrite concentrations (0, 25, 50, 100, 200, 400, and 800 mg NO_2_^−^/L) in 30 L acrylic square tanks, at a stocking density of approximately 29.1 kg/m^3^. During the 96 h exposure period, fish were not fed. The photoperiod was set to a 12 h light and 12 h dark cycle. During the experimental period, water quality parameters (water temperature, dissolved oxygen, pH, and salinity) were continuously monitored using a portable water quality analyzer (YSI Professional plus; YSI Inc., Yellow Springs, OH, USA). Additionally, levels of ammonia, nitrite, nitrate, and phosphate were measured following the Marine Environment Process Test Standard established by Ministry of Oceans and Fisheries (Table 1). Sodium nitrite (NaNO_2_, CAS No. 7632-00-0, Daejung Chemicals & Metals Co., Ltd., Siheung, Republic of Korea) was used to prepare a standard stock solution with a nitrite concentration of 30,000 mg NO_2_^−^/L. For each experimental tank, the nitrite concentrations were adjusted accordingly, and actual concentrations were verified once immediately after exposure using the same standard method (Table 2). To maintain consistent nitrite concentrations, rearing water was replaced daily, and nitrite was replenished accordingly. After the 96 h exposure period, blood samples were collected from all surviving fish after anesthesia with MS-222 (Sigma Chemical, St. Louis, MO, USA).

### 2.2. Lethal Concentration 50% (LC_50_)

The LC_50_ for waterborne nitrite was determined by monitoring fish survival in each tank at 0, 1, 3, 6, 12, 24, 48, 72, and 96 h after exposure, with dead individuals removed immediately upon detection. Based on the cumulative mortality after 96 h, the LC_50_ value was calculated using the probit model with a statistical software package (SPSS, version 29.0.2.0; SPSS Inc., Chicago, IL, USA).

### 2.3. Hematological Parameters

For hematological analysis, blood samples were collected from the caudal vein of all surviving individuals following nitrite exposure. Using syringes treated with heparin (Sigma Chemical, St. Louis, MO, USA), blood was drawn and immediately processed for hemoglobin levels, hematocrit, and red blood cell (RBC) counts. Hemoglobin was quantified using a clinical kit (Asan Pharm. Co., Ltd., Seoul, Republic of Korea) based on the cyan-methemoglobin method. Hematocrit levels were determined by filling microhematocrit capillary tubes with blood samples, which were then centrifuged at 15,536× *g* for 10 min using a microhematocrit centrifuge (Hematospin II, Hanil Scientific Inc., Gimpo, Republic of Korea), and measured with a microhematocrit reader (Hawksley & Sons Ltd., Lancing, UK). RBC count was performed after a 1:400 dilution of blood with Hayem’s solution, and the sample was analyzed with a hemocytometer (HSU-1401, Marienfeld Superior, Lauda-Königshofen, Germany) via an optical microscope (JSZ-6XN, Nanjing Jiangnan Novel Optics Co., Ltd., Nanjing, China).

### 2.4. Plasma Components

To investigate the effects of waterborne nitrite exposure on plasma components, blood samples were centrifuged (M15R, Hanil Scientific Inc., Gimpo, Republic of Korea) at 3000× *g* for 15 min at 4 °C to isolate plasma. All biochemical analyses were performed as described in [29]. Calcium (Ca^2+^) levels were determined using the orthocresolphthalein complexone (OCPC) method, and magnesium (Mg^2+^) concentrations were determined using a commercial kit (Asan Pharm. Co., Ltd., Seoul, Republic of Korea) following the Xylidyl blue-I method [29]. Glucose was quantified using the GOD/POD method with a clinical kit (Asan Pharm. Co., Ltd., Seoul, Republic of Korea), and cholesterol was assessed through a colorimetric assay [29]. Total protein was analyzed using the Biuret method [29]. Aspartate aminotransferase (AST) and alanine aminotransferase (ALT) activities were determined using the Reitman-Frankel method with a clinical kit (Asan Pharm. Co., Ltd., Seoul, Republic of Korea). Additionally, alkaline phosphatase (ALP) activities were measured using the King–King method [29].

### 2.5. Statistical Analysis Method

All surviving fish were included in the analysis, with three replicates conducted for each experimental group. All data points were presented in the graphs to clearly illustrate the distribution of the results. Statistical analysis was performed using one-way ANOVA followed by Tukey’s multiple comparison test to identify significant differences between the control and treatment groups. The analyses were conducted using GraphPad Prism version 10.4.1 (GraphPad Software, Boston, MA, USA), with statistical significance set at *p* < 0.05. Additionally, a principal component analysis (PCA) was performed to illustrate overall variation among the measured variables. Prior to PCA, data were standardized so that all variables received equal emphasis. Two principal components (PC1 and PC2) were then derived, and their respective explained variances were used to assess their contributions. All analyses were performed using Python (version 3.11.11) with the pandas, scikit-learn, and matplotlib libraries.

### 2.6. Approval of Animal Experimental Ethics

This experiment was conducted in accordance with the approval (2024-0032) from the Institutional Animal Care and Use Committee (IACUC) of Jeju National University, confirming that the experimental methods and procedures adhered to animal ethics guidelines. Fish that showed no response to even gentle external stimuli or exhibited complete loss of coordinated swimming ability were considered moribund and were euthanized with an overdose of MS-222 (500 mg/L). After the 96 h exposure period, all surviving fish were anesthetized with MS-222 (100 mg/L) for approximately 5 min, and a blood sample was collected from each individual. Upon completion of blood sampling, the fish were euthanized with MS-222 at 500 mg/L.

## 3. Results

### 3.1. Survival Rate and Lethal Concentration 50% (LC_50_)

**Figure 1:**
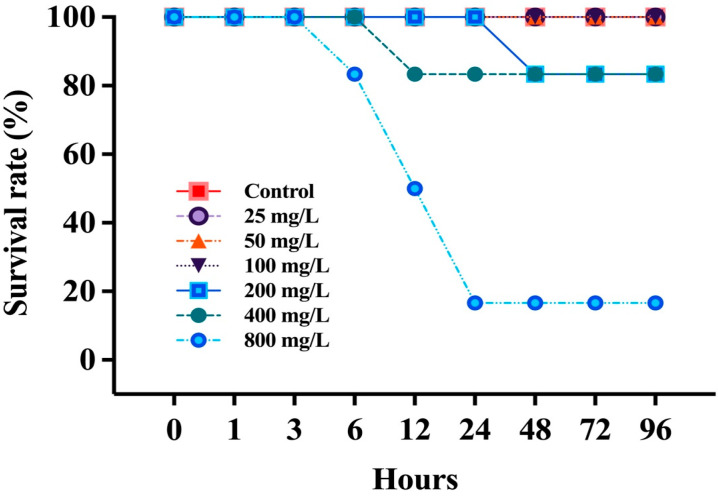
Survival rate of starry flounder, *Platichthys stellatus*, exposed to waterborne nitrite for 96 h.

The survival rate of *P. stellatus* according to waterborne nitrite exposure is shown in Figure 1. No dead individuals were found in exposures from control to 100 mg NO_2_^−^/L. At 200 mg NO_2_^−^/L, death occurred 48 h after exposure and 83.3% survived. At 400 mg NO_2_^−^/L, death occurred at 12 h of exposure, resulting in 83.3% survival. At 800 mg NO_2_^−^/L, death occurred at 6 h of exposure, resulting in a survival rate of 16.7%. Table 3 presents the LC_50_ value of *P. stellatus* under waterborne nitrite exposure. The LC_50_ was calculated as 574.47 mg NO_2_^−^/L.

### 3.2. Hematological Parameters

**Figure 2:**
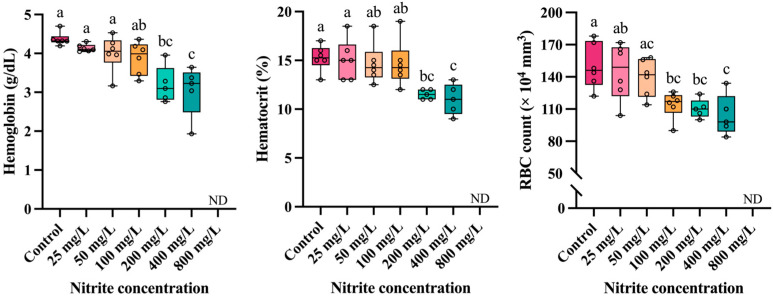
Hematological parameters of starry flounder, *Platichthys stellatus*, exposed to waterborne nitrite for 96 h. Different letters above the boxes indicate significant differences (*p* < 0.05) among treatments (one-way ANOVA followed by Tukey’s multiple comparison test).

The hematological parameters of *P. stellatus* according to waterborne nitrite exposure are shown in Figure 2. The hemoglobin concentration and hematocrit value of *P. stellatus* showed a significant decrease at concentrations above 200 mg NO_2_^−^/L (*p* < 0.05). RBC count showed a significant decrease at concentrations exceeding 100 mg NO_2_^−^/L (*p* < 0.05).

### 3.3. Plasma Components

**Figure 3:**
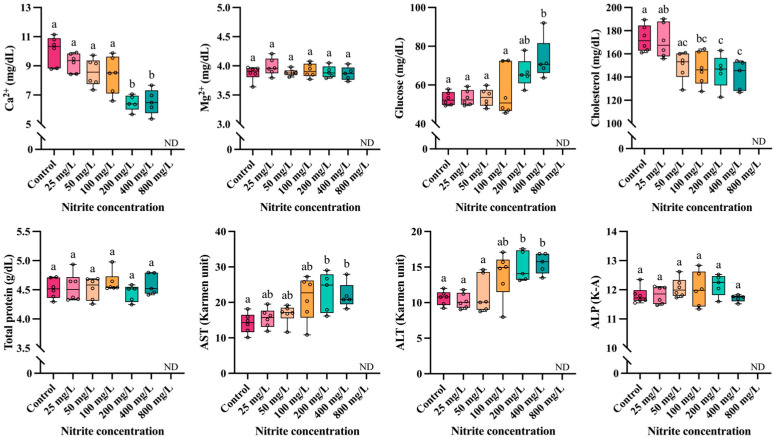
Plasma components of starry flounder, *Platichthys stellatus*, exposed to waterborne nitrite for 96 h. Different letters above the boxes indicate significant differences (*p* < 0.05) among treatments (one-way ANOVA followed by Tukey’s multiple comparison test).

The effects of waterborne nitrite exposure on the plasma inorganic components of *P. stellatus* are illustrated in Figure 3. Plasma calcium (Ca^2+^) levels showed a significant decrease at concentrations exceeding 200 mg NO_2_^−^/L (*p* < 0.05), whereas plasma magnesium (Mg^2+^) levels remained unchanged. Plasma glucose significantly increased at 400 mg NO_2_^−^/L (*p* < 0.05), while plasma cholesterol levels decreased significantly at concentration above 100 mg NO_2_^−^/L (*p* < 0.05). Total protein in plasma did not exhibit significant alterations. Aspartate aminotransferase (AST) and alanine aminotransferase (ALT) levels were significantly elevated at nitrite concentrations exceeding 200 mg NO_2_^−^/L (*p* < 0.05), whereas alkaline phosphatase (ALP) levels showed no significant changes.

### 3.4. Principal Component Analysis (PCA)

**Figure 4:**
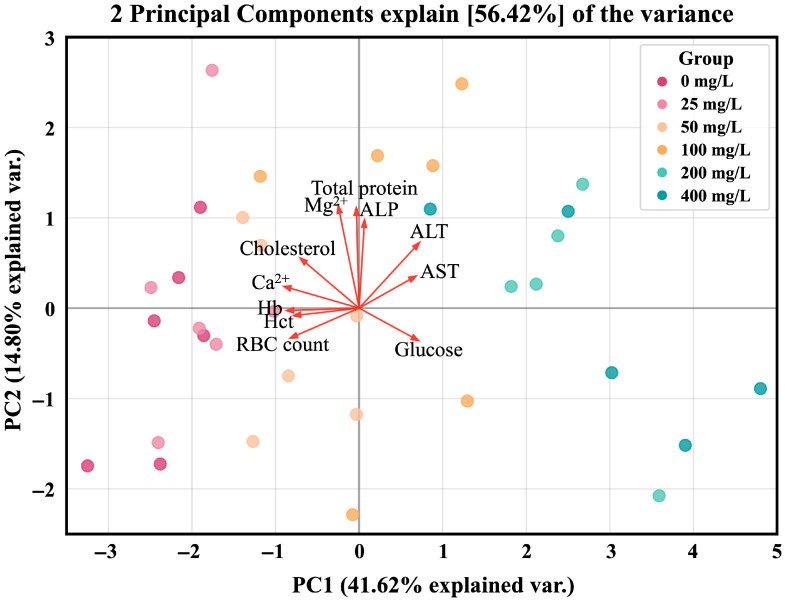
Principal component analysis (PCA) biplot of hematological parameters (Hb, Hct, RBC count) and plasma components (Ca^2+^, Mg^2+^, Glucose, Cholesterol, Total protein, AST, ALT, ALP). Hb, hemoglobin; Hct, hematocrit; AST, aspartate aminotransferase; ALT, alanine aminotransferase; ALP, alkaline phosphatase.

After 96 h of nitrite exposure, principal component analysis (PCA) revealed that PC1 and PC2 together explained 56.42% of the total variance (PC1: 41.62%, PC2: 14.8%) (Figure 4). The PCA score plot distinctly separated the control and low-concentration (25, 50, and 100 mg/L) groups from the high-concentration (200 and 400 mg/L) groups, suggesting that elevated nitrite concentrations induced marked physiological changes.

## 4. Discussion

High levels of nitrite exposure can be acutely toxic to fish, which can cause physiological disturbances in fish as well as mass mortality in severe cases; many researchers have reported lethal concentrations in various fish species following acute exposure to waterborne nitrite [30]. Ref. [31] reported that the 96 h LC_50_ of sea bream, *Sparus aurata* (weight: 1.1 g), exposed to nitrite were 1215, 2033, and 2648 mg/L, depending on salinity concentration (10, 20, and 30 psu), and they suggest that the effects of nitrite exposure among fish species may vary significantly through the unique nitrite regulatory mechanisms of each species. Ref. [32] suggest that nitrite exposure can oxidize hemoglobin in fish blood to methemoglobin, which can cause mortality with hypoxic symptoms (brown blood, hyperventilation, and gasping behavior) due to decreased oxygen-carrying capacity, and they reported that the LC_50_ of common snook, *Centropomus undecimalis* (weight: 1.26 g, length: 5.17 cm), exposed to nitrite was 285, 210, and 167 mg/L for 48, 72, and 96 h of exposure. Ref. [33] reported that the 96 h LC_50_ of rabbitfish, *Siganus rivulatus* (weight: 8.1 g), acutely exposed to nitrite was 345 mg/L, and in the high nitrite concentration range, fish showed general signs of stress, such as lethargy and darkening of body color. Ref. [34] reported that the 96 h LC_50_ of yellow catfish, *Pelteobagrus fulvidraco*, by acute exposure to nitrite was 226, 319, 439, and 644 mg/L, respectively, depending on size (0.034 g, 0.296 g, 3.52 g, 32.96 g), which means that the tolerance to nitrite exposure may vary depending on size. Ref. [35] reported that the 96 h LC_50_ of hybrid groupers, *Epinephelus lanceolatus* ♂ × *Epinephelus fuscoguttatus* ♀ (weight: 36.3 g, length: 13.0 cm), by acute exposure to nitrite was 856 mg/L, and they suggest that this was the result of hypoxia caused by the reduction of oxygen affinity in hemoglobin by converting Fe^2+^ to Fe^3+^ in the heme group of red blood cells by nitrite exposure. Ref. [36] reported that the LC_50_ of olive flounder, *Paralichthys olivaceus* (weight: 2.41 g, length: 7.29 cm), by nitrite exposure was 937, 872 and 768 mg/L at 48, 72 and 96 h of exposure, respectively, and they suggest that the mortality of fish by nitrite exposure was the result of neurological dysfunction due to potassium imbalance. In this study, the LC_50_ of starry flounders, *Platichthys stellatus*, was confirmed to be 574.47 mg NO_2_^−^/L by acute nitrite exposure for 96 h. This study establishes, for the first time, a species-specific LC_50_ reference value for nitrite in *P. stellatus*, providing a critical toxicological threshold for developing species-specific water quality criteria and optimizing nitrite management strategies in aquaculture. The 96 h LC_50_ value for *P. stellatus* was notably higher than those reported for other fish species, suggesting that *P. stellatus* exhibits a higher tolerance to nitrite. In the future, additional comprehensive studies on nitrite tolerance should be conducted through various studies, because the tolerance limit in fish exposed to nitrite can vary depending on not only the species but also the size, environmental conditions, and biological conditions.

Sublethal exposure to nitrite can directly affect the hematological parameters in fish, and hematological parameters are widely used as an important indicator of the toxic physiology in fish by exposure to toxic substances, as well as information on the health and physiological status in fish; a representative toxic effect of nitrite exposure is the conversion of hemoglobin to methemoglobin, which directly affects hematological properties [37,38]. In this study, high levels of nitrite (>100 or 200 mg/L) significantly decreased the hematological parameters such as hemoglobin concentration, hematocrit value, and RBC count of *P. stellatus*, which means that nitrite exposure can act as hematotoxicity in fish, thereby impairing oxygen supply/ventilation essential for survival. Ref. [39] reported that hemoglobin, hematocrit, and RBC count decreased in *Labeo rohita* exposed to nitrite; they suggested that the decrease in hemoglobin was due to methemoglobinemia caused by nitrite and hematopoietic dysfunction under hypoxic conditions, and the decrease in hematocrit and RBC count was attributable to RBC lysis. Likewise, reduction in hematological parameters has been reported in grass carp, *Ctenopharyngodon idella* [21], Nile tilapia, *Oreochromis niloticus* [40], turbot, *Scophthalmus maximus* [41] exposed to nitrite. Ref. [42] suggests that nitrite exposure may result in depletion of O_2_ content in the blood of fish due to a decrease in hemoglobin levels, and high methemoglobin levels may limit the β-adrenergic H^+^ extrusion from RBCs, inhibiting the ability of fish to respond to hypoxia. In addition, the increased activity of NADH-methemoglobin reductase to reconvert methemoglobin to hemoglobin under nitrite exposure may have caused more rapid destruction of RBCs by the spleen and kidney.

Nitrite enters the fish body through the Cl^−^/HCO_3_^−^ exchanger by competing with chloride ions, which can disrupt the ion balance in the body by changing the composition of plasma electrolytes [43,44]. Plasma inorganic components are essential biochemical biomarkers for assessing physiological damage and environmental stress in fish [45,46]. Ca^2+^ and Mg^2+^ are electrolytes abundant in fish plasma, and they have physiological and biochemical functions such as cell composition and osmoregulation [47]. In particular, Ca^2+^ maintains the structural integrity of the cell membrane and plays a key role in cell survival, including cell differentiation and apoptosis [48,49]. Ref. [50] reported that plasma Ca^2+^ levels decreased in *P. olivaceus* exposed to nitrite, and they suggest that high levels of nitrite exposure can disrupt ion homeostasis. Likewise, a decrease in Ca^2+^ levels was also observed in Atlantic sturgeon, *Acipenser oxyrinchus oxyrinchus* [51] and in *E. lanceolatus* ♂ × *E. fuscoguttatus* ♀ [35]. In the present study, the plasma Ca^2+^ level of *P. stellatus* significantly decreased due to nitrite exposure at high concentrations (>200 mg/L), suggesting that nitrite-induced hypoxia negatively affects Ca^2+^ channel activity, thus disrupting calcium homeostasis and decreasing extracellular Ca^2+^ levels. Meanwhile, Mg^2+^ serves as a cofactor for ATPases involved in ion pumps and exchangers, playing crucial roles in neurotransmission, muscle contraction, and nucleic acid synthesis [52,53]. Ref. [54] reported that plasma Mg^2+^ levels in *O. niloticus* significantly decreased during the recovery period after nitrite exposure, implying that nitrite-induced renal damage may result in Mg^2+^ malabsorption. In this study, no significant changes in plasma Mg^2+^ levels of *P. stellatus* were observed. Further studies should investigate, at the molecular level, the mechanisms underlying nitrite-induced disturbances in ion balance. Particular emphasis should be placed on exposure duration, species-specific ion regulation capacity, and the regulatory pathways of Ca^2+^ and Mg^2+^ metabolism to provide a more comprehensive understanding of nitrite toxicity in aquatic organisms.

Plasma glucose represents the energy metabolism of fish, and it is also a common stress indicator in fish exposed to toxicity [55]. Refs. [44,56] reported that the plasma glucose level of *O. niloticus* significantly increased due to nitrite exposure, which suggested that the energy demand increased due to metabolic defense against nitrite stress. Ref. [39] reported that nitrite exposure significantly increased plasma glucose levels in *L. rohita*, and they suggest that stress caused by nitrite exposure induce catecholamine secretion increase and glycogenolysis activation, which increased blood sugar levels. In the present study, plasma glucose levels in *P. stellatus* were significantly elevated following exposure to nitrite at concentrations over 400 mg/L, which is thought to be due to enhanced gluconeogenesis to supply glucose for the NADH-methemoglobin reductase pathway involved in methemoglobin reduction.

Plasma cholesterol plays a major role in cell membrane composition, molecular recognition and signaling processes, and it is also used as an indicator of lipid metabolism in fish [57]. Ref. [9] reported that plasma cholesterol in *P. fulvidraco* significantly decreased due to nitrite exposure, suggesting the possibility of efficient use of carbohydrates as an energy source in stress situations. Ref. [58] also reported that cholesterol concentration in Japanese pufferfish, *Takifugu rubripes*, significantly decreased due to nitrite exposure, suggesting that nitrite exposure can interfere with lipid metabolism. Ref. [59] also reported a significant decrease in plasma cholesterol in Large yellow croaker, *Larimichthys crocea* due to nitrite exposure, and which was the result of lipids being used as an energy source due to nitrite stress. In this study, plasma cholesterol significantly decreased due to exposure to nitrite (>100 mg/L), which is thought to be the result of liver damage caused by nitrite exposure, decreased lipid excretion from liver cells and accumulation of cholesterol in the liver.

Total plasma protein is involved in blood osmotic regulation, pH maintenance, and humoral immune responses, and it is used as a major indicator for evaluating the health status and protein metabolism of fish [60]. Ref. [61] reported a significant decrease in blood protein levels in dark-banded rockfish, *Sebastes inermis* by nitrite exposure. They suggest that the increased energy demand in fish exposed to nitrite stimulates protein catabolism, and renal damage caused by nitrite exposure may decrease plasma protein through renal excretion. Ref. [62] reported that plasma protein of clown knifefish, *Chitala ornata* significantly decreased due to nitrite exposure, which was due to the disruption of blood electrolyte–water balance due to impaired active ion absorption. Ref. [33] also reported that nitrite exposure significantly decreased the plasma total protein level of *S. rivulatus*, which was the result of plasma dilution and protein degradation caused by nitrite exposure. However, in this study, nitrite exposure did not significantly change the plasma total protein level of *P. stellatus*, which means that nitrite exposure had a limited effect on protein metabolism in *P. stellatus*.

Plasma enzyme components such as AST, ALT, and ALP are used as sensitive indicators to evaluate liver damage in fish due to environmental stress [63]. Ref. [64] reported that plasma AST and ALT activities of *T. rubripes* significantly increased due to nitrite exposure, which was a result of increased synthesis of transaminase required for induction of gluconeogenesis due to nitrite stress and inflow into the blood due to liver tissue damage. Ref. [65] reported significant increases in AST, ALT, and ALP in *S. maximus* by nitrite exposure, which suggested that AST, ALT, and ALP were released into the bloodstream due to hepatic necrosis and liver tissue damage caused by nitrite exposure. Ref. [11] also reported that plasma AST, ALT, and ALP were significantly increased in pacamã, *Lophiosilurus alexandri* by nitrite exposure, which was a result of liver damage caused by nitrite stress. Ref. [66] reported a significant increase in the AST and ALT of *E. lanceolatus* ♂ × *E. fuscoguttatus* ♀ exposed to nitrite. They argued that nitrite disrupted nitrogen metabolism in fish, and transaminase activity increased as an adaptive response to regulate glutamate and protein synthesis in the body. In this study, exposure to high levels of nitrite (>200 mg/L) significantly increased plasma AST and ALT levels in *P. stellatus*. It is believed that tissue hypoxia occurred due to the conversion of hemoglobin to methemoglobin due to nitrite toxicity, and that the synthesis of AST and ALT enzymes required for the gluconeogenesis process increased by the anaerobic metabolism activation and the gluconeogenesis process promotion in this hypoxic state. Meanwhile, in the present study, no significant variation in the plasma ALP level of *P. stellatus* was observed under nitrite exposure. Furthermore, hematological parameters and plasma components suggest that nitrite exposure disrupts physiological systems in *P. stellatus*. Principal component analysis (PCA) provided an integrated overview, clearly separating low- and high-concentration groups and indicating that alterations in blood and plasma variables reflect nitrite-induced stress.

## 5. Conclusions

In conclusion, high-concentration waterborne nitrite exposure (>200 mg NO_2_^−^/L) induced mortality in *P. stellatus* (16.7% mortality at 200 and 400 mg NO_2_^−^/L, 83.3% mortality at 800 mg NO_2_^−^/L), with an LC_50_ value of 574.47 mg NO_2_^−^/L. Hematological parameters, including hemoglobin, hematocrit, and RBC count, were significantly decreased following nitrite exposure. Plasma Ca^2+^ and cholesterol levels significantly decreased, and plasma glucose, AST, and ALT levels significantly increased. Principal component analysis (PCA) further revealed clear separation between low- and high-concentration groups, underscoring pronounced physiological changes induced by nitrite toxicity. These findings highlight that high-level nitrite exposure (>100 or 200 mg NO_2_^−^/L) can cause both mortality and significant hematological disruptions in *P. stellatus*. Further studies should investigate the combined effects of nitrite and other environmental stressors commonly present in aquaculture settings, as such co-exposures are likely under real farming conditions. In addition, molecular-level studies on the mechanisms underlying disturbances in nitrogen metabolism and ionic homeostasis will provide a deeper understanding of nitrite toxicity in aquatic organisms.

## Figures and Tables

**Table 1:** The chemical components of water and experimental condition used in the experiments.

Item	Value
Temperature (°C)	16.8 ± 0.29
pH	8.11 ± 0.03
Dissolved Oxygen (mg/L)	6.31 ± 0.22
Salinity (‰)	30.21 ± 0.1
Ammonia (µg/L)	3.78 ± 0.45
Nitrite (µg/L)	3.59 ± 0.11
Nitrate (mg/L)	0.23 ± 0.04
Phosphate (µg/L)	8.37 ± 0.42

**Table 2:** Measured waterborne nitrite concentrations (mg NO_2_^−^/L) for each treatment group. Values represent concentrations measured immediately after exposure.

Nitrite Concentration (mg NO_2_^−^/L)							
Nitrite concentrations	Control	25	50	100	200	400	800
Measured nitrite concentrations	0.01	26.4	53.2	108.4	209.4	416.8	823.6

**Table 3:** Lethal concentration (LC_50_) of starry flounder, *Platichthys stellatus*, exposed to waterborne nitrite for 96 h.

95% Confidence Limits	
Probability	Estimate (mg/L)
0.01	27.19
0.10	272.98
0.20	376.48
0.30	451.11
0.40	514.87
0.50	574.47
0.60	634.07
0.70	697.84
0.80	772.47
0.90	875.96
0.99	1121.75

## Data Availability

Data are contained within the article.
